# The Central New York Homœopathic Society and Its Protest against Eclecticism

**Published:** 1883-12

**Authors:** E. B. Nash


### THE CENTRAL NEW YORK HOMŒOPATHIC SOCIETY AND ITS PROTEST AGAINST ECLECTICISM.

*To the Editor of* THE HOMŒOPATHIC PHYSICIAN:

DEAR SIR:—Will you kindly allow me space in your columns to answer an editorial in the *Hahnemannian Monthly,* entitled "The Protest"? My reason for asking this favor of you is the following:

In the *Hahnemannian Monthly*, 1882, p. 543, Dr. F. F. Laird published a case of prosopalgia cured by Hypericum. The paper ended with a contemptible fling at those whom he is pleased to style the "anti-pathologists."

Thinking this slur to be an unjust reflection on those who regard *symptomatology* as the best-known basis for prescribing homœopathically, I criticised it by asking four questions. In the next number of the journal appeared my criticism, followed immediately by Dr. Laird's reply (showing how eager the editor was to give Dr. Laird undue advantage).

Thinking I would at least have an opportunity to defend my side of the question (symptomatology), I wrote a very careful reply, subjecting it to the criticism of some professional brethren, who pronounced it both courteous and good. It was sent to the editor of the *Hahnemannian Monthly.* I waited long and patiently, but my article did not appear. I therefore wrote to Dr. Farrington. He replied that he had given it to Dr. Dudley. Wrote to Dr. Laird; answered he would see about it. Heard nothing more; so wrote to Dr. Dudley, and received curt reply, saying, in effect, that my article was not worthy of publication.

This dealing, unjust and ungentlemanly as it is, leaves us exposed to comment and criticism, but allows no opportunity for defense. The editor may reply that my article was not worth space in his precious pages; but that was my affair—always supposing the tone of the article to be properly courteous; the weakness and frailty of the argument are my fault and misfortune. This, then, is my reason for asking for space in your pages.

THE PROTEST.

In referring to the meeting of the Central New York Homœopathic Society which criticised his eclectic resolution, the editor (of the *H. M.*) says: "Only sixteen members were present." "No comments." Of course not! Comments would show the disparity of numbers between the membership of the Central Society and the Institute. Also might bring to notice the fact that the resolutions ("the protest") were passed by the Central in full meeting while the sixteen were present, and not (as in case of Institute) by trickery, after a majority had gone home. No comments!!

Now as to Syphilinum. In the first place, Dr. Schmitt proposed that this substance be proven (why did not this *fair-minded* editor mention this ?), and all the experience given at the meeting—much abbreviated in the report—was more for the purpose of showing that its occasional empirical use showed it to be an agent worthy of a careful *proving* than to teach its empirical use, isopathically or any other way. Not only this, but the discussion as to the use of Syphilinum was brought out by a question from the President: Has any one had any experience with it? Moreover, the general verdict of the Society was that *nosodes should be proven.* This and the tenor of the discussion showed that the Central Society was not advocating empiricism; rather the contrary—that unproven drugs should not be used. A fair and impartial critic would have noted this.

"They thought it was Homœopathy," says this editor. How does he know what they *thought?* Did they *say so?* Perhaps they considered it isopathy. [Or perhaps *he* thought the Central was discussing eclectic methods, as does the Institute.—Editor H. P.]

The true inwardness of the editorial is, we believe, this: The Central Society dared to enter its protest—*as homœopathists,* not as members of the Institute—against the eclectic resolution of said American Institute. As the Institute is supposed to represent Homœopathy in America—and so all adherents of that school, members and non-members, in this country—the Central Society had the best of rights, that of *self-defense,* for denouncing the false position taken by the Institute. E. B. Nash, M. D.

